# Semi-allogeneic vaccine for T-cell lymphoma

**DOI:** 10.1186/1479-5876-5-39

**Published:** 2007-08-08

**Authors:** Jin Yu, Mark S Kindy, Sebastiano Gattoni-Celli

**Affiliations:** 1 Department of Neurosciences, Medical University of South Carolina, Charleston, SC, USA; 2 Department of Radiation Oncology, Medical University of South Carolina, Charleston, SC, USA; 3 Ralph H. Johnson VA Medical Center, Charleston, SC, USA

## Abstract

**Background:**

Experimental results from studies with inbred mice and their syngeneic tumors indicated that the inoculation of semi-allogeneic cell hybrids (derived from the fusion between syngeneic tumor cells and an allogeneic cell line) protects the animal host from a subsequent lethal challenge with unmodified syngeneic tumor cells.

**Methods:**

Semi-allogeneic somatic cell hybrids were generated by the fusion of EL-4 T lymphoma cells (H-2^b^) and BALB/c-derived renal adenocarcinoma RAG cells (H-2^d^). Cell hybrids were injected intra-peritoneally (i.p.) in C57BL/6 mice (H-2^b^) before challenging the mice with a tumorigenic dose of EL-4 cells.

**Results:**

Semi-allogeneic tumor cell hybrids could not form a tumor in the animal host because they expressed allogeneic determinants (H-2^d^) and were rejected as a transplant. However, they conferred protection against a tumorigenic challenge of EL-4 cells compared to control mice that were mock-vaccinated with i.p.-injected phosphate-buffered saline (PBS) and in which EL-4 lymphomas grew rapidly to a large size in the peritoneal cavity. Screening of spleen-derived RNA by means of focused microarray technology revealed up-regulation of genes involved in the Th-1-type immune response and in the activation of dendritic antigen-presenting cells (APC).

**Conclusion:**

The results of our studies are entirely consistent with the concept that CD80- and CD86-expressing APC play a central role in mediating the immune protection induced by semi-allogeneic vaccines by activating a Th-1 response and instructing T cells responsible for killing autologous tumor cells.

## Background

The capacity of T cells to recognize allogeneic MHC molecules as intact structures on the surface of foreign cells is called direct T-cell allorecognition and is responsible for the powerful immune reactions associated with transplant rejection, a phenomenon called "alloagression". To a large extent this is due to the ability of allogeneic stimulation to mobilize up to 10% of all T lymphocytes, compared with a precursor T-cell frequency of between 10^-4 ^and 10^-5 ^for most common antigens. At the same time, each of the lymphocytes activated through direct allorecognition will also recognize a specific antigenic peptide presented in the context of a self major histocompatibility complex (MHC) molecule (MHC restriction). Cross-reactivity between alloantigens and self MHC-restricted antigens can be harnessed to target tumor antigens [1].

Experimental results from studies with inbred mice and their syngeneic tumors indicated that the inoculation of semi-allogeneic cell hybrids (derived from the fusion between syngeneic tumor cells and an allogeneic cell line) protects the animal host from a subsequent lethal challenge with unmodified syngeneic tumor cells [2,3]. Later studies confirmed the validity of these observations by showing that the adoptive transfer of immunity induced by semi-allogeneic tumor cells required T lymphocytes and that the enhanced immunity was not due simply to an allogeneic effect. In fact, co-administration (injection) into experimental mice of allogeneic cells together with irradiated autologous tumor cells (i.e., without fusion) did not fully protect them from a subsequent challenge with autologous tumor cells, supporting the conclusion that, in order to achieve maximum anti-tumor protection, the tumor-associated antigen and the alloantigen needed to be on the same cell [4-8].

We reported on the use of semi-allogeneic vaccines as stimulators of HIV-envelope-specific cytotoxic T lymphocytes (CTL) and we proposed that semi-allogeneic cell hybrids functionally mimic APC by concomitantly stimulating alloantigen-specific T helper cells via allogeneic MHC, and antigen-specific CTL precursors via antigen presentation through self-MHC [9]. We also proposed that the Th-1 cytokine response, induced through alloantigen-specific help, activates more efficiently antigen-specific CTL and that the cytokine-rich microenvironment of allograft rejection is crucial to attracting dendritic APC.

Recognition of tumor antigens, generally weakly immunogenic, can be enhanced greatly by semi-allogeneic vaccines. Consistent with this possibility is the demonstration that vaccination of mice with allogeneic fibroblasts transfected with DNA from mouse melanoma cells and expressing a single MHC class I syngeneic determinant, was capable of protecting mice against a tumorigenic challenge with syngeneic melanoma cells [10]. Similarly, the immunogenic properties of tumor cells expressing transfected allogeneic determinants are consistent with this interpretation [11,12].

Although the immunogenic properties of somatic cell hybrids made with dendritic cells have been the object of intense study [13-15], it is important to realize that generation of semi-allogeneic dendritic cell vaccines is very labor-intensive and difficult to implement in a clinical setting. The studies described in this report indicate that effective semi-allogeneic vaccines do not require dendritic cells as a fusion partner, as they specifically activate the host dendritic cells.

## Methods

### Cell lines

RAG cells are a non-reverting, 8-azaguanine-resistant clone of the Renal-2a cell line, originally derived from a kidney adenocarcinoma of a BALB/c mouse (H-2^d ^haplotype). RAG cells are deficient in the X-linked hypoxanthine-guanine phosphoribosyl transferase gene (HGPRT^-^); therefore, they are killed in culture media containing a supplement of hypoxanthine, aminopterin, and thymidine (HAT). We confirmed that RAG cells are non-reverting, 8-azaguanine-resistant and HAT-sensitive before using them in experiments of cell fusion. These cells grow as a monolayer. EL-4 cells were established from a T-cell lymphoma induced in a C57BL mouse (H-2^b ^haplotype) by the chemical carcinogen 9,10-dimethyl-1,2-benzanthracene. These cells grow in suspension. RAG and EL-4 cell lines were purchased from the American Type Culture Collection (ATCC). Both cell lines were propagated in Dulbecco's modified Eagle's medium (DMEM) supplemented with 10% fetal bovine serum (FBS), glutamax and antibiotics (Gibco/Invitrogen).

### Derivation of semi-allogeneic hybrids

RAG cell monolayers were trypsinized and the resulting single-cell suspensions were counted and checked for viability using the Trypan Blue exclusion test. RAG cells in suspension were mixed with EL-4 cells at a 1 : 3 ratio in serum-free DMEM, containing 50 μM sodium dodecyl sulfate (SDS), and spun down at 300×g for 4 min at room temperature. The mixed cell pellet was then slowly resuspended in 1 mL 50% polyethylene glycol (PEG)-1450 (cell-culture grade from the ATCC and diluted in serum-free DMEM) over a one-minute period while gently stirring. The cell suspension was then slowly diluted over a two-minute period with DMEM supplemented with 10% of fetal bovine serum (FBS). After washing twice in DMEM + 10% FBS, fused cells were plated in selective medium (DMEM + 10% FBS and HAT supplement) [16]. Under these culture conditions only RAG × EL-4 semi-allogeneic somatic cell hybrids will survive, since RAG cells are killed and EL-4 cells are lost because they grow in suspension and do not attach to the plastic substrate like somatic cell hybrids do.

### In vivo animal studies

Pathogen-free C57BL/6 male mice were obtained through the Jackson Laboratories (Bar Harbor, ME). All mice were housed in the VA animal facility located on the seventh floor of the Strom Thurmond Biomedical Research Bldg. Ten-week-old C57BL/6 male mice were injected intraperitoneally (i.p.) with 1 × 10^6 ^RAG × EL-4 semi-allogeneic somatic cell hybrids in 0.5 mL phosphate-buffered saline (PBS). As a control, age-matched mice were either mock-vaccinated i.p. with 0.5 mL PBS or injected i.p. with 1 × 10^6 ^RAG cells in 0.5 mL PBS. In separate experiments utilizing 1 × 10^6 ^EL-4 cells as an antigen, with or without 1 × 10^6 ^RAG cells, in 0.5 mL PBS, all cells used as vaccines were lethally irradiated with 30 Gy (3,000 rad) in a ^137^Cs irradiator. Three weeks after vaccination or mock-vaccination each mouse was challenged by i.p. injection with either 2 × 10^4 ^or 3 × 10^4 ^EL-4 lymphoma cells in 0.5 mL PBS. Mice were then monitored very closely for growth of i.p. tumors and sacrificed when the abdomen became clearly extended, generally within three weeks. Necropsy was performed on each animal and EL-4 abdominal tumors were carefully dissected and weighed out. Animal studies were conducted according to internationally recognized ethical guidelines and under the full approval of the Charleston VA Medical Center Institutional Animal Care & Use Committee (IACUC) (protocol ACORP#346).

### Histology

Tumor tissue specimens were fixed in 4% paraformaldehyde (PFA) in PBS for 1–2 days, before being transferred to 30% sucrose in 4%PFA solution until the tissue samples sank to the bottom of the tube. Specimens were then placed into labeled plastic molds, covered with OCT (Optimal Cutting Temperature), and frozen by contact with dry ice. Frozen sections (5 μm thick) were placed on to slides coated with a solution containing 1% gelatin and 0.2% chromium poatassium sulfate; slides were dried overnight. The following day tissue sections on slides were stained with Hematoxylin & Eosin and a coverslip was mounted (Permount) on each slide.

### Statistical considerations

Data were analyzed by one-way analysis of variance for analyses of statistical significance, with p < 0.05 indicating statistical significance, using GraphPad Prism software program.

### Studies with focused microarrays

Total RNAs were isolated from spleens of untreated mice, mock-vaccinated and vaccinated mice, respectively. Spleens collected from mice vaccinated with semi-allogeneic cell hybrids were from animals that had no tumor. These pooled RNAs were subjected to focused microarray analyses of murine Th1-Th2-Th3 immune function (Superarray Bioscience Corp., cat.# OMM-034).

Subsequently, RNAs were also analyzed using dendritic cell (DC)-specific microarrays (Superarray Bioscience Corp., cat.# OMM-406). All data from these experiments were normalized using minimum value background subtraction and expression of housekeeping genes, before being compared. Minimum value is the lowest density reading of a spot on the array. It is defined, by default, as the background and it is subtracted from the intensity value for each spot on the array. Scatter plot comparisons were performed in a blinded fashion by the microarray manufacturer (Superarray Bioscience Corp.), and included only genes that showed either up-regulation or down-regulation by 1.5 fold or more.

## Results

### In vivo animal studies with EL-4 T lymphoma cells

**Figure 1:**
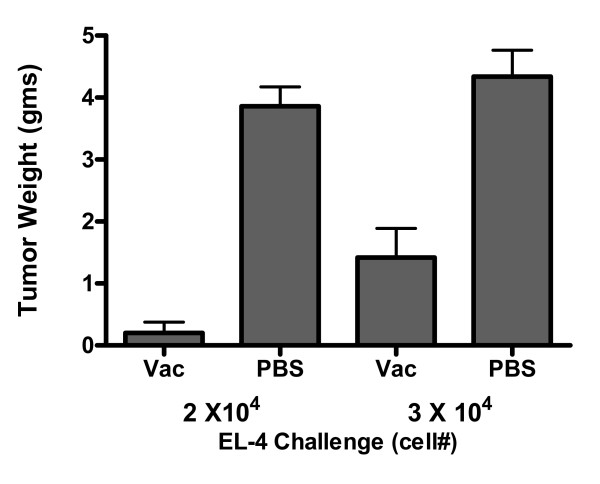
Anti-tumor protection conferred by semi-allogeneic vaccines. The bar graph shows the average tumor weight per mouse and the standard deviation. Differences in tumor weight between mock-vaccinated mice (PBS) and mice vaccinated with semi-allogeneic cell hybrids (Vac) were statistically significant for both challenges: p-values were < 0.0001 and 0.0017 for the 2 × 10^4 ^and the 3 × 10^4 ^EL-4 lymphoma cells challenge, respectively.

These experiments were carried out to investigate whether RAG × EL-4 semi-allogeneic somatic cell hybrids were able to protect C57BL/6 mice against a tumorigenic challenge with EL-4 cells. Ten-week-old C57BL/6 male mice were injected intraperitoneally (i.p.) with 1 × 10^6 ^RAG × EL-4 semi-allogeneic somatic cell hybrids in 0.5 mL phosphate-buffered saline (PBS). As a control, age-matched mice were mock-vaccinated i.p. with 0.5 mL PBS. Three weeks after vaccination or mock-vaccination each mouse was challenged by i.p. injection with either 2 × 10^4 ^or 3 × 10^4 ^EL-4 lymphoma cells in 0.5 mL PBS. Mice were then monitored very closely for growth of i.p. tumors and sacrificed when the abdomen became clearly extended, generally within three weeks. Necropsy was performed on each animal and EL-4 abdominal tumors were carefully dissected and weighed out. Figure 1 shows the result of these experiments.

**Figure 2:**
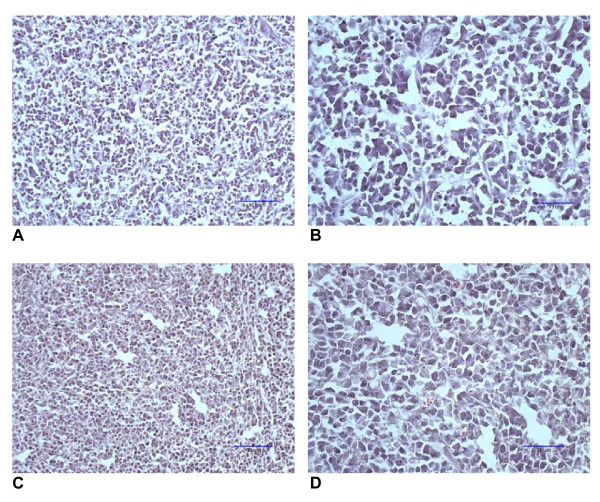
Hematoxylin & Eosin staining of tissue sections from EL-4-derived tumors. Panels A and B show low and high magnification (with scale bars) of a typical EL-4 tumor from a mouse mock-vaccinated with PBS three weeks before challenge. Panels C and D show low and high magnification (with scale bars) of EL-4 tumor sections from a mouse vaccinated with RAG × EL-4 semi-allogeneic hybrids three weeks before challenge. Note, that both tumors appear to be poorly differentiated (anaplastic), and that there are no morphologic features that distinguish them.

All mock-vaccinated mice challenged with EL-4 cells developed large tumors. The mean tumor size of mice injected i.p. with 2 × 10^4 ^cells was 3.9 grams and the mean tumor size of mice injected i.p. with 3 × 10^4 ^cells was 4.3 grams. In contrast, three of five vaccinated mice that were challenged i.p. with 2 × 10^4 ^EL-4 cells showed no evidence of abdominal tumor growth at time of sacrifice and the mean tumor size in this group of mice was 0.2 grams. Similarly, one of five vaccinated mice that were challenged i.p. with 3 × 10^4 ^EL-4 cells showed no evidence of abdominal tumor growth at time of sacrifice and the mean tumor size in this group of mice was 1.4 grams. Differences in tumor size between vaccinated and mock-vaccinated mice were statistically significant for both challenges (p-values were < 0.0001 and 0.0017 for the 2 × 10^4 ^and the 3 × 10^4 ^EL-4 lymphoma cells challenge, respectively). Histological examination of tumors from mice mock-vaccinated with PBS compared to those from mice vaccinated with RAG × EL-4 semi-allogeneic somatic cell hybrids showed no notable difference (Figure 2).

**Figure 3:**
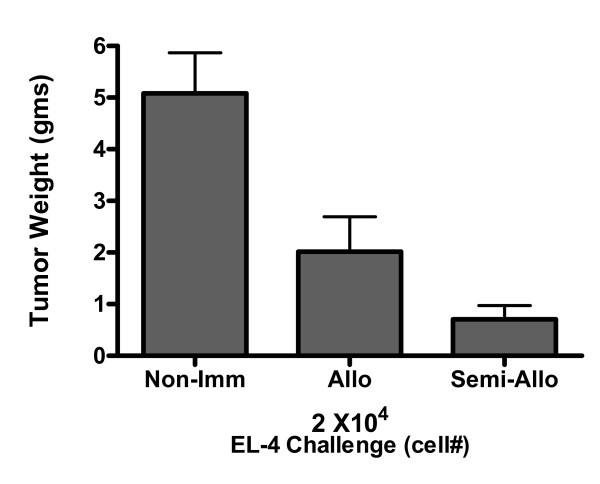
Anti-tumor protection conferred by semi-allogeneic vs. allogeneic vaccines. The bar graph shows the average tumor weight per mouse and the standard deviation. Differences in tumor weight between mock-vaccinated mice (Non-Imm) and mice vaccinated with semi-allogeneic cell hybrids (Semi-Allo) were the most significant (p-value < 0.0001). Also significant were differences in tumor weight between mock-vaccinated mice and mice vaccinated with allogeneic RAG cells (p-value = 0.0183), as well as between mice injected with semi-allogeneic vs. allogeneic vaccines (p-value = 0.0458).

A second set of animal experiments was performed to investigate whether the anti-tumor protection was the result of an allogeneic effect. Three groups of mice were utilized: 1) mock-vaccinated with 0.5 mL PBS alone (Non-Imm); 2) vaccinated with 1 × 10^6 ^RAG cells in 0.5 mL PBS (Allo); 3) vaccinated with 1 × 10^6 ^RAG × EL-4 semi-allogeneic somatic cell hybrids in 0.5 mL PBS (Semi-Allo). After three weeks each mouse was challenged by i.p. injection with 2 × 10^4 ^EL-4 lymphoma cells in 0.5 mL PBS and mock-vaccinated mice showed an extended abdomen because of i.p. tumor growth within three weeks from the day of challenge. At the time of sacrifice, all mock-vaccinated mice challenged with EL-4 cells had developed large tumors and the mean tumor size was 5.1 grams; also, four of five mice vaccinated with allogeneic RAG cells and challenged with EL-4 cells had i.p. tumors (mean tumor size: 2.0 grams). In contrast, four of ten mice vaccinated with RAG × EL-4 semi-allogeneic somatic cell hybrids and subsequently challenged i.p. with 2 × 10^4 ^EL-4 cells showed no evidence of abdominal tumor growth at time of sacrifice and mean tumor size in this group of mice was 0.7 grams (Figure 3). The results of these animal studies clearly indicate that, although the i.p. injection of allogeneic cells (RAG) conferred some degree of anti-tumor protection compared to mock-vaccinated mice, semi-allogeneic vaccines were significantly better at inducing specific anti-tumor protection.

**Figure 4:**
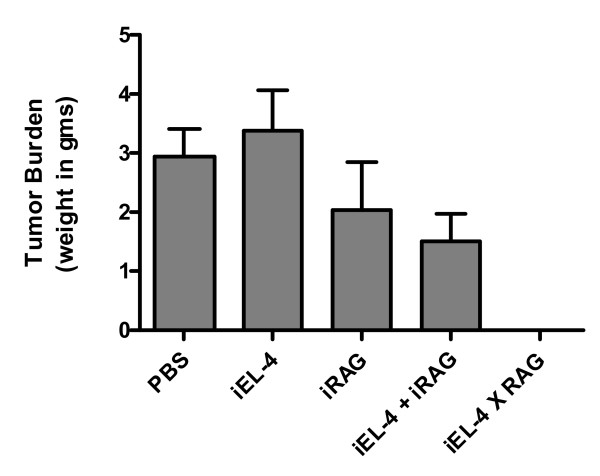
Anti-tumor protection conferred by injection of irradiated semi-allogeneic vaccines compared to co-injection of irradiated EL-4 cells plus irradiated RAG cells. The bar graph shows the average tumor weight per mouse and the standard deviation for each group of mice. The five groups were immunized as follows: 1) mock-vaccination (PBS); 2) 1 × 10^6 ^irradiated EL-4 cells (iEL-4); 3) 1 × 10^6 ^irradiated RAG cells (iRAG); 4) 1 × 10^6 ^irradiated EL-4 cells co-injected with 1 × 10^6 ^irradiated RAG cells (iEL-4 + iRAG); 5) 1 × 10^6 ^irradiated EL-4 × RAG semi-allogeneic somatic cell hybrids (iEL-4 × RAG). Using mock-vaccination (PBS) as a control, the p-values were as follows: iEL-4 p-value = 0.5469; iRAG p-value = 0.3065; iEL-4 + iRAG (co-injection) p-value = 0.0417; and iEL-4 × RAG (hybrids) p-value < 0.00001.

A third set of animal experiments was performed to investigate whether i.p. vaccination with 1 × 10^6 ^irradiated EL-4 cells plus 1 × 10^6 ^irradiated RAG cells would protect the mice from a subsequent challenge with 2 × 10^4 ^EL-4 live cells. Lethal irradiation (30 Gy in a ^137^Cs irradiator) was needed to prevent the growth of live EL-4 cells as a tumor. We also wanted to compare the anti-tumor protection resulting from co-injection of EL-4 and RAG irradiated cells with the anti-tumor protection observed following vaccination with 1 × 10^6 ^RAG × EL-4 irradiated semi-allogeneic somatic cell hybrids. For these experiments five groups of mice were injected i.p. as follows: 1) mock-vaccination with 0.5 mL PBS/mouse; 2) vaccination with 1 × 10^6 ^irradiated EL-4 cells in 0.5 mL PBS/mouse; 3) vaccination with 1 × 10^6 ^irradiated RAG cells in 0.5 mL PBS/mouse; 4) vaccination with 1 × 10^6 ^irradiated EL-4 cells plus 1 × 10^6 ^irradiated RAG cells in 0.5 mL PBS/mouse; 5) vaccination with 1 × 10^6 ^RAG × EL-4 irradiated semi-allogeneic somatic cell hybrids in 0.5 mL PBS/mouse. After three weeks each mouse was challenged by i.p. injection with 2 × 10^4 ^live EL-4 lymphoma cells in 0.5 mL PBS. Nineteen days after challenge, all mice mock-vaccinated with PBS and all mice vaccinated with irradiated EL-4 cells had developed tumors, and most of the mice vaccinated with irradiated RAG cells or with irradiated EL-4 cells plus irradiated RAG cells had also developed tumors. In contrast, none of the mice vaccinated with RAG × EL-4 irradiated semi-allogeneic somatic cell hybrids showed any signs of tumor growth at day nineteen after challenge (Figure 4).

Later on some of these mice developed intra-peritoneal tumors; however, four of ten mice vaccinated with RAG × EL-4 irradiated semi-allogeneic somatic cell hybrids remained tumor-free at five weeks after challenge. Even though the co-injection of irradiated EL-4 cells and irradiated RAG cells seemed to confer a significant degree of anti-tumor protection compared to mock-vaccinated (PBS) mice, these experimental results clearly demonstrate that, in order to achieve maximum anti-tumor protection, the tumor-associated antigen and the alloantigen need to be on the same cell (the hybrid).

### Studies with focused microarrays

To investigate the biological and molecular requirements for protective immunity and understand the mechanisms underlying the specific anti-tumor response induced by semi-allogeneic vaccines, total RNA was isolated from spleens of untreated mice, mock-vaccinated and vaccinated mice and analyzed by microarray technology.

Spleen RNA from both experiments was subjected to focused microarray analyses of murine Th1-Th2-Th3 immune function (Superarray Bioscience Corp., cat.# OMM-034). The results obtained with both sets of samples showed consistent over-expression of select genes associated with Th-1 immune function in the spleen of mice vaccinated with semi-allogeneic somatic cell hybrids (immune mice). In contrast, the spleen from mock-vaccinated (non-immune) mice revealed over-expression of Th-2 immune functions. The spleen of mice immunized with allogeneic vaccine (allo-immune mice) over-expressed some – but not all – of the transcripts that were elevated in the spleen of immune mice. The results of these microarray studies are summarized in Tables 1 and 2.

**Table 1:** Genes over-expressed in spleen of *Immune Mice*. Values within square brackets [] represent the fold increase in the expression of each gene by mice vaccinated with semi-allogeneic vaccines compared to mice mock-vaccinated with PBS.

**CD80 (B7-1) **[1.9] **CD86 (B7-2) **[1.8]	Cell surface molecules expressed by activated APC that provide a co-stimulatory signal to T cells by interacting with CD28
**C2ta **[2.0]	Enhancer of T-cell activation and proliferation and suppressor of Th2-type cytokine expression
**STAT1 **[2.0]	Signal transducer and activator of transcription 1, activated by various ligands including interferon α (IFN- α) and IFN-γ
**STAT4 **[2.0]	Another member of the Stat family of transcription factors that is essential for mediating responses to IL-12 in lymphocytes and for regulating the differentiation of T helper cells
**CD40 **[2.0]	A co-stimulatory molecule expressed on the surface of B lymphocytes, dendritic cells, and follicular dendritic cells
**FASLG **[1.8]	Critical for triggering apoptosis of some types of cells following interaction with FAS
**HAVCR2 **[2.5]	Hepatitis A virus cellular receptor 2, over-expressed by activated CD4 Th1 cells and CD11b+ macrophages

**Table 2:** Genes over-expressed in spleen of *Non-Immune Mice*. Values within square brackets [] represent the fold increase in the expression of each gene by mice mock-vaccinated with PBS compared to mice vaccinated with semi-allogeneic vaccines.

**LAT **[2.9]	Linker for activation of T cells, involved in T-cell receptor (TCR)-initiated, T cell-specific signaling events and possibly associated with over- stimulation and apoptosis of T cells
**GATA3 **[2.5]	GATA binding protein 3, a transcription factor that favors expression of Th2-type cytokines
**SOCS3 **[1.5]	Suppressor of cytokine signaling 3, is a negative regulator of cytokine signaling
**Gfi1 **[3.8]	Growth factor independent 1, a transcriptional repressor essential during myeloid differentiation (Gfi1-/- mice exhibit diminished monocyte-derived dendritic cells and disturbed cytokine production by macrophages in response to LPS)

Mice immunized with allogeneic vaccine (allo-immune mice) shared with the immune mice the over-expression of only some of the genes (C2ta, Stat1, FASLG, and HAVCR2) listed in Table 1, indicating that the immune mechanisms triggered by the allogeneic response do not account for the molecular complexity and specificity of the anti-tumor immune protection conferred by semi-allogeneic vaccination.

These observations prompted us to analyze the same RNAs using dendritic cell (DC)-specific microarrays (Superarray Bioscience Corp., cat.# OMM-406). When compared to mock-vaccinated (non-immune) and to allogeneic cell-vaccinated (allo-immune) mice, the spleen of immune, tumor-free mice (vaccinated with semi-allogeneic somatic cell hybrids) expressed significantly higher levels of the following notable genes (in addition to those listed in Table 1): CD209a (DC-SIGN), CD28, CD83, FAS, Flt3L, H2-DMa, ICOSL (B7-H2), ITGAX (CD11C), LTA, NFKB, and XCL1 (ATAC). These results are summarized in Table 3.

**Table 3:** DC-specific genes that are over-expressed in spleen of I*mmune Mice*. Values within square brackets [] represent the fold increase in the expression of each gene by mice vaccinated with semi-allogeneic vaccines compared to mice mock-vaccinated with PBS. These over-expressed genes are in addition to those listed in Table 1.

**DC-SIGN **[4.0] **(CD209a)**	A cell surface C-type lectin expressed by dendritic cells that mediates antigen capture for processing and presentation
**CD28 **[1.6]	T cell-specific receptor for CD80 and CD86
**CD83 **[1.9]	The most reliable activation marker for mature dendritic cells
**FAS **[2.1]	The apoptosis-mediating membrane-associated polypeptide that belongs to the tumor necrosis factor receptor superfamily
**Flt3L **[1.7]	A ligand that expands type-1 dendritic cells and enhances the immune response by augmenting T-cell activity
**H2-DMa **[2.0]	Involved in antigen processing and presentation of exogenous peptide antigens via MHC class II
**ICOSL **[2.6] **(B7-H2)**	Is a ligand for T cell-specific, cell surface receptor ICOS and acts as a co-stimulatory signal for T-cell proliferation and cytokine secretion
**CD11c **[1.7] **(ITGAX)**	Expressed by dendritic cells that tend to generate Th1 effectors
**LTA **[1.7] **(TNF-β)**	Lymphotoxin α or tumor necrosis factor β
**NFKB **[1.5]	A transcription factor required for STAT-4 expression during dendritic cell maturation
**XCL1 **[2.5] **(ATAC)**	An activation-induced, T cell-derived and chemokine-related type 1 cytokine, that cooperates with interferon (IFN)-γ in the up-regulation of CD40 and interleukin (IL)-12 expression

The results of these microarray experiments indicate that semi-allogeneic vaccines trigger the recruitment and activation of helper T lymphocytes and dendritic APC; in turn, these cells establish a strong Th-1 response, which leads to the activation of CTL to specifically recognize and kill their target tumor cells.

## Discussion

These studies were undertaken to further our understanding of the mechanisms underlying the specific anti-tumor response induced by semi-allogeneic vaccines. Specifically, the results of our microarray studies are entirely consistent with the concept that CD80- and CD86-expressing APC play a central role in mediating the immune protection induced by semi-allogeneic vaccines by activating a strong Th-1 response and instructing T cells responsible for killing autologous tumor cells. These results are also consistent with a recent report on molecular signatures induced by interleukin (IL)-2 on human peripheral blood mononuclear cells [17].

Interest in semi-allogeneic cancer vaccines has been increasing, as demonstrated by the publication of successful pre-clinical and clinical studies by us [9,16,18,19] and others [13,20] that validate this approach. Specifically, we observed that lymphocytes from HLA-A2+ melanoma and HIV+ patients, stimulated in vitro with HLA-A2-restricted antigenic peptides in the presence of irradiated semi-allogeneic cell hybrids lysed target cells presenting the same peptide more efficiently than lymphocytes stimulated with antigenic peptide alone [9,19]. We were allowed by the FDA, under BB-IND 6266, to conduct two Phase I studies in patients with disseminated melanoma [16] and metastatic adenocarcinoma [18]. We determined that treatment of cancer patients with irradiated semi-allogeneic vaccines is associated with minimal or no toxicity and can induce a specific anti-tumor immune response, measured by a positive delayed-type hypersensitivity (DTH) to irradiated autologous tumor cells injected intra-dermally. In one melanoma patient with stage III disease and treated with semi-allogeneic vaccine, we observed the reduction in size of two lymph nodes with melanoma cells and the patient is still free of disease almost ten years after vaccination.

However, in spite of some encouraging outcomes, the results of clinical studies employing semi-allogeneic vaccines as immunotherapeutics against a variety of neoplasias have not matched the expectations raised from the striking anti-tumor protection conferred by semi-allogeneic vaccines in a variety of animal models. It is tempting to speculate that the well-documented success of semi-allogeneic vaccines in animal models is due to the high prevalence of cancer stem cells [21] in the experimental tumors utilized for these studies. We observed that 2 × 10^4 ^EL-4 T lymphoma cells injected i.p. into C57BL/6 mice give rise to a 5 gram tumor in three weeks or less, suggesting that a large percentage of these cells fulfill the operational criteria for cancer stem cells.

There is a growing consensus that malignancies remain incurable unless their cancer stem cells are eliminated [21]. It is reasonable to explore the concept that clinically effective semi-allogeneic vaccines need to be generated from patient-derived cancer stem cells fused with appropriate allogeneic cells, because only cancer stem cells will express the pathogenetic and antigenic determinants that are critical to a successful immunotherapy. Cancer stem cells are responsible for the persistence/recurrence of tumors; these cells tend to be drug-resistant and are responsible for the dissemination and metastatic growth of tumors. Cancer stem cells must be killed to achieve clinical responses that are both complete and durable. Semi-allogeneic vaccines derived from the fusion of cancer stem cells and an appropriate allogeneic cell partner would provide an additional treatment for cancer patients, especially before their disease reaches the late and often irreversible stage. Therefore, it is virtually certain that the field of immunotherapy will benefit considerably from the rapidly increasing body of knowledge being generated in the emerging field of human cancer stem cell research.

## Authors' contributions

JY was responsible for conducting the animal experiments, MSK designed the animal experiments and performed the statistical analysis, and SGC was responsible for overall experimental design and wrote the manuscript. All authors read and approved the final manuscript.
